# Recent Progress of Cement-Based Materials Modified by Graphene and Its Derivatives

**DOI:** 10.3390/ma16103783

**Published:** 2023-05-17

**Authors:** Houxuan Li, Ge Zhao, Hong Zhang

**Affiliations:** 1 School of Civil Engineering, Chongqing Jiaotong University, Chongqing 400074, China; 2 Maintenance Inspection Department of Chongqing Yuhe Expressway Co., Ltd., Chongqing 400799, China

**Keywords:** cement-based materials, concrete, graphene, graphene derivatives

## Abstract

Graphene, with its excellent properties and unique structure, has been extensively studied in the context of modifiable cement-based materials. However, a systematic summary of the status of numerous experimental results and applications is lacking. Therefore, this paper reviews the graphene materials that improve the properties of cement-based materials, including workability, mechanical properties, and durability. The influence of graphene material properties, mass ratio, and curing time on the mechanical properties and durability of concrete is discussed. Furthermore, graphene’s applications in improving interfacial adhesion, enhancing electrical and thermal conductivity of concrete, absorbing heavy metal ions, and collecting building energy are introduced. Finally, the existing issues in current study are analyzed, and the future development trends are foreseen.

## 1. Introduction

Cement-based materials are widely used in infrastructure construction due to their high production efficiency, abundant raw material sources and good workability. As a result, they are the most commonly used construction materials in the world [1]. However, the production of Portland cement is an industrial activity with high energy consumption and pollution. According to relevant data, the cement industry’s carbon dioxide emissions account for approximately 7% of the total global carbon emissions [2], while energy consumption represents about 2% of the global total [3,4,5]. The significant greenhouse gas emissions generated during cement production undeniably affect the environment. In addition, conventional cement-based composite materials exhibit typical brittle material properties due to their poor crack resistance, low tensile strength, and limited strain capacity. These limitations make conventional cement-based materials unable to meet the diverse usage requirements in various engineering fields.

**Figure 1:**
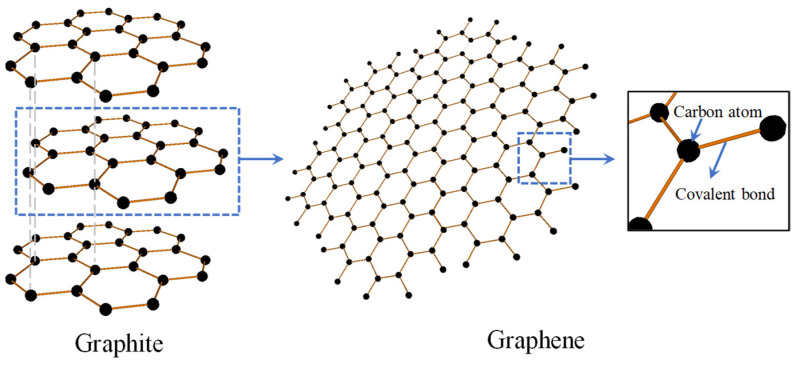
Schematic diagram of graphene structure.

With the rapid development of nanomaterial science, researchers have used nanomaterials combined with industrial solid waste materials (such as fly ash [6], plastic [7], waste glass [8], etc.) to replace a portion of cement in concrete. This approach reduces material costs, reduces carbon emissions and ensures concrete strength, providing a new pathway for research into green and sustainable concrete. In addition, the development of nanoparticles has opened up new avenues for researchers. Nanomaterials have qualities such as nano-filling, surface effect, and macroscopic quantum tunneling effect, which can improve the overall mechanical properties and durability of conventional cement-based materials [9]. For example, adding nano silica particles into cement mortar can increase the compressive strength of concrete by about 70% [10]. Likewise, adding 5% alumina nanoparticles resulted in a 43% increase in the elastic modulus of cementitious composites [11,12,13]. Furthermore, adding carbon nanotubes [14,15] can improve the frost resistance of concrete. In recent years, the application of graphene materials in Portland cement has attracted much attention [16]. Graphene materials can be divided into graphene and graphene derivatives, including graphene oxide (GO), reduced graphene oxide (RGO), graphene nanosheets (GNPs), graphene sulfide, etc. As shown in Figure 1, graphite, commonly seen in everyday life, consists of stacked graphene layers, a two-dimensional honeycomb lattice structure formed by tightly bonded carbon atoms. Weak van der Waals forces hold adjacent layers together. When graphite is stripped down to a single layer, it becomes graphene, which is just one carbon atom thick.

Since Novoselov [17] successfully broke through the graphene separation method in 2004, researchers have extensively explored the preparation method [16], structural properties [1], and practical application of graphene [18,19]. Novoselov used a viscous substance to strip graphene from graphite. This method is relatively simple, but produces only small sheets of graphene and is inefficient. In addition, the thermal spallation graphene oxide method is a simple and cost-effective method for graphene production, which has great potential for large-scale graphene preparation. However, because the sample structure [20], temperature and gaseous environments [21] will affect the production quality of GO materials, it is necessary to accurately control and optimize these factors. The focus of research on graphene oxide has shifted from the production of graphene to the controlled preparation of graphene. By oxidizing graphite with substances such as nitric acid, sulfuric acid, and potassium permanganate, and then reducing or thermally treating it, graphene oxide can be obtained [22]. It is difficult to improve the preparation efficiency of the above methods. Recently, a novel methodology for the development of a versatile electrochemically active platform based on freestanding graphite nanoplatelets (GNP) has been developed by exploiting the interiors of hollow carbon nanofibers (CNF), comprising nanographene stacks using dry ball-milling. This method is expected to achieve efficient production and precise control of graphene materials [23].

**Figure 2:**
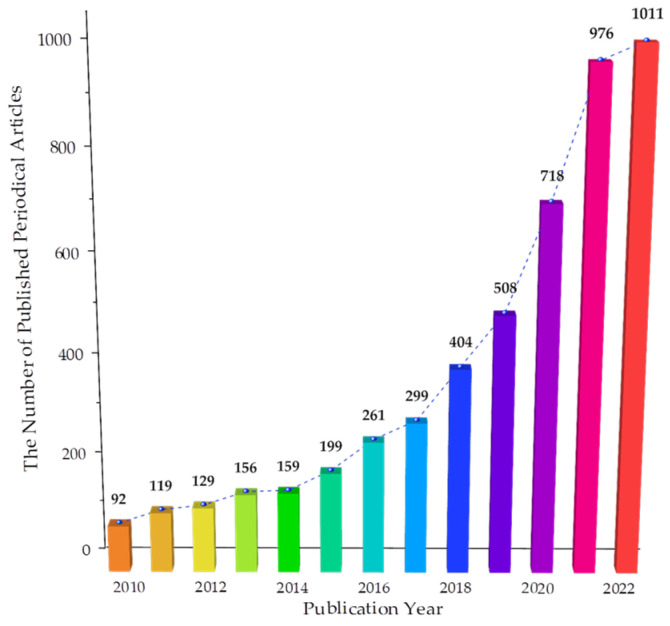
Number of papers published on graphene and cement-based materials.

Researchers found that graphene materials have a large specific surface area [24], excellent electrical and thermal conductivity [25,26], and excellent mechanical properties [27,28]. In addition, graphene derivatives contain many active functional groups such as carboxyl, hydroxyl and epoxy groups. These groups can form chemical bonds with the clinker in Portland cement, resulting in a strong bond between graphene and Portland cement. The unique physical and chemical properties make graphene materials stand out in improving the properties of Portland cement. As shown in Figure 2, this paper searched the Web of Science website for graphene concrete papers from 2010 to 2022 and found a large increase in the number of studies in recent years. Many scholars have investigated the incorporation of graphene materials into concrete, which is no longer limited to improving mechanical properties, but its durability [29], electrical conductivity [30], thermal conductivity, pressure sensitivity [31], thermal energy absorption, and other aspects have been explored in depth.

However, there is a lack of systematic classification and summarization in the context of graphene-modified cementitious materials. Therefore, in the Section 2, Section 3 and Section 4 of this paper, we comprehensively describe the progress in applying graphene to improve the mechanical properties, working properties, and durability of cement-based materials. Then, in Section 5, we analyze the chemical mechanisms of graphene materials in enhancing concrete properties and present the current application status of special concrete. Finally, we summarize research issues and provide prospects for research development trends. It is hoped that this article can provide a reference for future research.

## 2. Workability

The workability of cemented materials directly affects the strength and durability of concrete. It is a composite property that includes rheology, contractility, thermal diffusion, and other aspects. In the following, we elaborate on the application of graphene materials from these aspects. The following paper will elaborate on the application of graphene materials in these aspects.

Many researchers have demonstrated that as the amount of GO added to cement composites increases, the fluidity of the mixture decreases. This is due to the minimal particle size of GO, which enables it to fill the gaps in the cemented material. In addition, the large specific surface area of GO absorbs a large amount of free water [32] and Portland cement [33], leading to an increase in the viscosity of the matrix [34] and a decrease in fluidity. Therefore, when adding graphene materials, water reducing agent should be appropriately added to reduce slump loss, but this will increase the cost of concrete [1]. In this paper, we propose that the fluidity reduction in cement-based materials is also related to the functional group on the surface of graphene. A large number of active groups such as hydroxyl (–OH), carboxyl (–COOH), and epoxy (–O–) groups in the graphene dispersion react with water, which will adsorb part of the free water in the clean slurry mix for flow, resulting in the lack of lubrication within the cementation materials. As a result, graphene reduces the fluidity of concrete.

Based on the ability of GO to adsorb free water, Jia et al. [35] developed the GO anti-shrinkage agent to promote the silicate reaction in cement. Meanwhile, the thermal conductivity of GO enables the heat to be fully transferred, accelerating the hydration speed and finally forming a dense structure in the hydration process of cement, which inhibits the dry shrinkage cracks of concrete [36]. In addition, Chen et al. [37] proved that GO can also reduce the creep coefficient of concrete. Finally, Chu et al. [38] focused on the heat diffusion properties of graphene materials. They found that incorporating graphene sulfonates also significantly reduced the ablative rate of concrete, which was considered for use in constructing nuclear power plants.

## 3. Mechanical Properties

Although numerous experimental data have shown that graphene materials can improve the mechanical properties of cement-based materials, experimental results have varied due to differences in the amount, structure, and dispersion of graphene impurity. Therefore, the following will provide a summary and analysis from two perspectives: the macroscopic and microscopic reinforcement mechanisms of graphene materials, and the factors affecting the performance enhancement of silicate concrete.

### 3.1. Enhancement Mechanism of Graphene Materials

Graphene has exceptionally high strength and stiffness, and when uniformly dispersed in concrete, it forms denser concrete structures, reducing the number and size of micro-defects in concrete. Researchers have found that the tensile and compressive performance of concrete can be improved by adding a moderate amount of graphene powder [39], single-layer graphene sheet powder [40], or graphene dispersion [41]. Additionally, the van der Waals forces between graphene and cement gel can reduce the distance between cement particles, facilitating complete hydration reactions and improving concrete’s overall strength [42].

**Figure 3:**
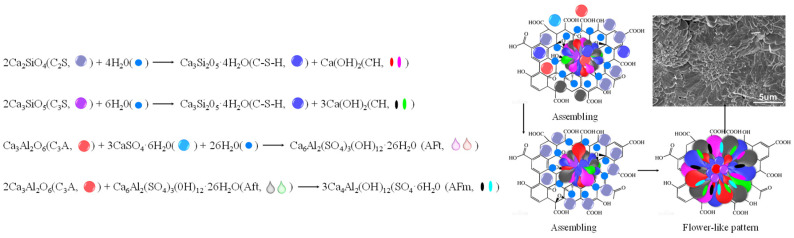
Schematic diagram and SEM image of cement hydration reaction and product [44].

From the microscopic point of view, when GO is involved in hydration reactions, hydrated crystals will form dense, regular, and blooming flower-like structures. Once it encounters pores, cracks, or loose structures, it can be dispersed into flower-like crystals, which fill holes and cracks [43]. Lv et al. [44] clarified the formation mechanism of the “flower-like” microstructure of hydration crystals, as shown in Figure 3. He believes that when GO meets cement, chemical groups such as –OH, –O– and –COOH on the surface preferentially react with tricalcium silicate (C3S), dicalcium silicate (C2S), and tricalcium aluminate (C3A) to form flower-like hydration products. The hydration products are made up of Aft (Aft), AFm (AFm), calcium hydroxide (C–H), and calcium silicate hydrate (C–S–H). The shape of these hydrated crystals is controlled by graphene oxide. Once these hydrated crystals encounter pores, cracks, or loose structures, they grow apart to form flower-like compact structures, acting as a filler and a crack inhibitor. In addition, Luo [45] and Wu [46] believed that the functional groups of GO caused the “mechanical interlocking” effect inside the cement material, which increased the microscopic friction between the material surface and the cement base, and formed a solid interfacial force inside the cement-based material. These ideas are the direct reason why graphene improves the mechanical properties of cement-based materials.

### 3.2. Influencing Factors of Concrete Performance Enhancement

In this paper, we summarize the results of the mechanical property tests performed on the graphene-modified concrete and organize them in Table 1. The test specimen used for data collection was plain Portland cement concrete, which is composed of plain Portland cement, a polycarboxylate-based water reduction admixture, coarse aggregate, fine aggregate, and water. Each test group used ultrasonic or mechanical dispersion methods to disperse the graphene material in water. Subsequently, the graphene dispersion liquids were added to other admixtures for stirring, casting, and forming. Due to the diverse production methods of graphene materials, we discuss as many physical properties as possible in this paper.

It can be seen from Table 1 that the addition of an appropriate amount of graphene materials has improved the mechanical properties of concrete. Different blending contents of graphene materials will lead to different strengthening effects. Lv [47] and Cao [48] believed that when a small amount of GO is added, the mechanical properties of concrete will increase with the increase of GO. Still, after a certain amount, the high specific surface area of the GO appears to have an agglomerative effect, which decreases the compactness of the concrete. As a result, the enhancement effect on the mechanical properties will be reduced.

In the data collected, compared with ordinary concrete in the control group, when GO with a mass proportion of 0.02%, a thickness of 15 nm, an average size of 80~300 nm, and oxygen content of 25.45% was added, the compressive strength and bending strength of concrete after 28 days of curing reached their maximum values, with an increase of 60.1% and 84.5% respectively [49]. When GO with a mass proportion of 0.03%, a thickness of less than 8 nm, a size of about 80~260 nm, and an oxygen content of 29.75% was added, the bending strength of concrete specimens after curing for 28 days was increased by 60.7% at the maximum [48]. When adding graphene with a mass ratio of 0.05%, an average thickness of 8 nm, and a particle size of 550 nm, the elastic modulus of the concrete specimen after 14 days of curing was increased by 18.07% [50].

Due to the influence of curing time, the oxygen content, diameter, thickness, and lifting efficiency of different tests are also different. However, it can be seen from the references [51,52] that the increase of concrete compressive strength gradually decreases with the increase in curing time. This is because the main product of the hydration reaction in concrete is calcium silicate hydrate gel, which is the primary source of early strength in concrete. Therefore, the addition of graphene material can promote the hydration reaction and accelerate the formation of gel formation, thus increasing the early strength of the concrete. Similar views were also proposed in reference [53]. However, after 28 days of curing, the growth rate of the concrete slowed. Therefore, the strengthening effect of the graphene material is relatively small, and the increase in strength of the concrete strength is also reduced. In addition, some scholars believe that microwave curing can improve the enhancement effect of GO on the mechanical properties of concrete [54], but the curing cost of this method needs to be considered.

The lamellar diameter, specific surface area, and oxygen content can affect the dispersion of graphene in cemented materials and further affect the efficiency of graphene-based materials in improving their mechanical properties. The smaller diameter of graphene flakes in cement-based composites leads to higher dispersion, hydration degree, and crystallinity enhancement efficiency, resulting in increased strength of the composites [55]. In addition, the larger specific surface area can also increase the contact area between solvent molecules and GO surface, enhance the interaction force between cement-based materials and GO, and reduce the dispersion of graphene materials in water [56]. The higher oxygen content of GO indicates more functional groups on its surface, which will improve its dispersibility and solubility in concrete [49]. Moreover, the dispersion of GO is also related to its charge properties in the solvent. As the oxygen content increases, the number of negatively charged sites on its surface increases, leading to enhanced interactions with positively charged ions or molecules, which helps to improve the graphene dispersion.

**Figure 4:**
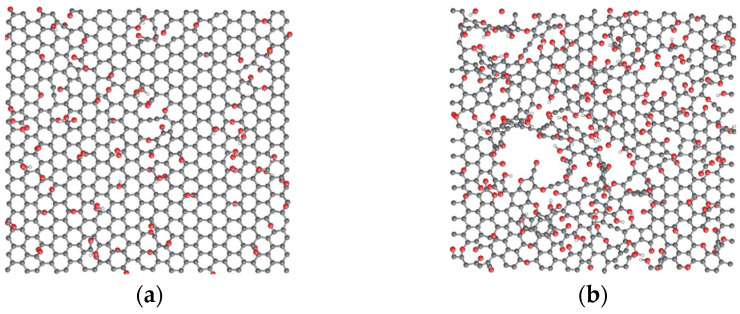
Morphologies of GO with different oxygen content: (**a**) morphology of GO with 20% oxygen content and (**b**) morphology of GO with 33% oxygen content [16]. Carbon, oxygen, and hydrogen atoms are gray, red, and white, respectively.

As can be seen from the table, the cement-based enhancement effect of ordinary graphene materials is lower than the GO enhancement for the common graphene material. This is because ordinary graphene can quickly produce an agglomerative phenomena after incorporating a cement base. In contrast, GO has a relatively good dispersion due to many oxygen-containing functional groups. Moreover, when GO is incorporated into the cement matrix, it acts as a template and bridging agent in cement hydration crystals, promoting cement hydration and regulating the hydration of the crystals. As a result, the microstructure of the cement hydrated crystal is denser and the macroscopic properties of the cement matrix are improved. Figure 4 shows the morphology of two graphene materials. GO contains many hydroxyl, epoxide, and carbonyl functional groups, which facilitate the dispersion of GO in water and widen the distance between graphene layers, quickly shedding water to produce a stable dispersion composed of a single sheet [16,19]. In conclusion, improving the dispersion of graphene materials is a prerequisite for enhancing the performance of concrete.

**Table 1:** Summary of the effects of different properties and mass ratios of graphene materials on the mechanical properties of ordinary Portland cement concrete.

Mechanical Property	Mechanical Property Enhancement Value	Properties and Mass Fraction of Graphene and Its Derivatives						Reference
		Type	Thickness/nm	Layered Diameter/nm	Specific Surface Area/m^2^g^−1^	Oxygen Content	Mass Content	
Compressive strength	The tensile strength of the specimens cured for 28 days increased by 78.6%.	GO	15	80~300	/	25.45%	0.02%	[49]
	The compressive strength of specimens cured for 7, 14, and 28 days increased by 33.15%, 10.88%, and 17.72%, respectively		3.4~7	10~50	100~300	/	0.03%	[51]
	The compressive strength of the specimens cured for 14 days increased by 11.05%		/	40~80	40~300	30.7%	0.05%	[50]
	The compressive strength of the specimens cured for 28 days increased by 47.2%		~8	80~260	/	29.75%	0.05%	[48]
	The compressive strength of the specimens cured for 28 days increased by 30.64%		/	500~10,000	/	40~50%	0.07%	[57]
	The compressive strength of the specimens cured for 7 and 28 days increased by 58.2% and 47.7%, respectively		0.8~2	5000~10,000	/	26.54%	0.15%	[52]
	The compressive strength of the specimens cured for 28 days increased by 22.7%	GNPs	4~20	5000~10,000	/	/	0.03%	[58]
	The compressive strength of the specimens cured for 28 days increased by 33.9%		5	6800	/	/	0.04%	[59]
	The compressive strength increased by 28% after 27 days of curing in saturated lime water		8	550	/	/	0.05%	[60]
Flexural strength	The flexural strength of the specimens cured for 28 days increased by 84.5%	GO	15	80~300	/	25.45%	0.02%	[49]
	The flexural strength of the specimen cured for 28 days increased by 29.75%		8~10	/	600~700	/	0.02%	[47]
	The flexural strength of the specimens cured for 28 days increased by 60.7%		8	80~260	/	29.75%	0.03%	[48]
	The flexural strength of the specimen cured in saturated lime water for 27 days increased by 28%	GNPs	8	550	/	/	0.05%	[60]
Elastic modulus	Elastic modulus increased by 9% after curing in saturated lime water for 27 days	GNPs	8	550	/	/	0.05%	[60]
	Elastic modulus increased by 18.07% after 14 days of curing	GO	/	40~80	40~300	30.7%	0.05%	[50]

## 4. Durability

The application and impact of graphene materials on concrete’s durability are described below. We discuss the effects of graphene materials on concrete ranging from impermeability, chloride ion erosion resistance, freeze–thaw resistance, carbonization resistance, fatigue properties, abrasion resistance, crackle repair, and antimicrobial properties to provide a reference for the design and application of graphene-modified cement-based materials.

### 4.1. Impermeability

The impermeability of cement-based materials is the main factor affecting their durability. There are two main research directions to improve permeability: surface coating with graphene materials and improving the pore structure inside the material. Regarding surface coating, A. Haibnejad [61] found that direct coating of GO material on the concrete surface could reduce water and capillary absorption of concrete by about 40% and 57%, respectively. Tan [62] used graphene to modify traditional epoxy coatings to improve the impermeability of concrete. On the other hand, Gao [63] proposed that when the optimal dosage of GO-modified polyurethane coating is 0.5%, too little material will lead to poor coating density and uneven dispersion.

On the other hand, too much material can lead to material accumulation. Therefore, researchers have also used graphene to modify isobutyl triethoxysilane [64], silane and isopropyl alcohol [65], polyacrylate emulsion [66], and other materials. The waterproof effect of the modified coating is significantly improved and the cost of such coatings is low. Therefore, this method has a tremendous economic advantage in improving the impermeability of concrete.

### 4.2. Resistance to Chloride Ion Erosion

**Figure 5:**
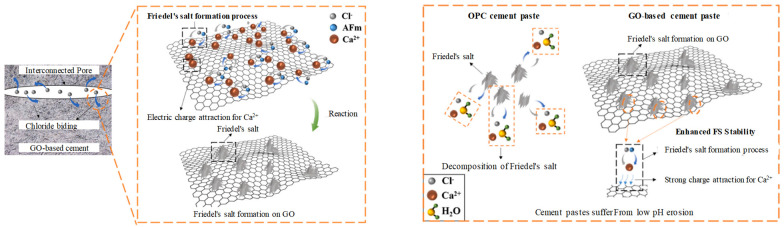
Theoretical diagram of graphene oxide binding to chloride ions [69].

In contrast to the coating method, direct doping of graphene materials can demonstrate the impermeability and overall strength of cemented materials and prevent the erasure of chloride ions and the rusting of internal steel rods. Regarding microstructure and permeability resistance theory, Krystek et al. [67] attributed the chloride ion resistance to the shrinkage of large capillary pores. Similarly, Zhou et al. [68] found through simulation that the incorporation of graphene materials led to the remodeling of microstructure and inhibited the migration of water in the pores of cement slurry. However, Wang [53] and Long [69] added another critical reason why graphene materials could improve the chloride ion erosion resistance of concrete, as shown in Figure 5: They suggest that the chemical crosslinking effect between divalent cations (such as calcium ions) and surface functional groups of GO promotes the formation of Friedel’s salts (F salts). As a result, the chloride ion binding capacity of cementitious material is enhanced. In addition, GO exhibits strong charge adsorption on calcium ions, effectively inhibiting F salt’s decomposition and making its structure more stable. This finding suggests that the chloride-curing properties of graphene-modified concrete can help to suppress chloride ion erosion.

Regarding the blending content of graphene materials, Li [70] found that using graphene nanosheets with 0.06 wt% content to modify cement mortar is beneficial to hinder the diffusion of chloride ions inside the cement matrix, which can achieve the best densification effect. Du [71] believed 1.5 wt% of graphene A3775 was the best mixing amount. At this content, the water permeability, the chloride ion diffusion coefficient, and the chloride ion migration coefficient were reduced by 80%, 80%, and 40%, respectively. Zhou [72] added the effects of water–cement ratio, fly ash, and load on the chloride ion resistance of cementitious materials.

### 4.3. Freeze–Thaw Resistance

In concrete structures in alpine regions, cyclic freeze–thaw is a widespread and severe disease that leads to the expansion of internal pores and microstructural damage, thus significantly affecting the performance and lifetime of the concrete. Blending graphene materials can change the porosity inside the concrete and indirectly improve its freeze–thaw resistance. Xu and Fan [73] investigated the effect of different graphene contents on the frost resistance of concrete and proposed that the optimal amount of GO was 0.03%. At this dose, the specimen showed a 34.83% increase in compressive strength compared to the fiducial specimen after 200 times of salt freeze-out, with the lowest loss rates in mass and dynamic elastic modulus.

In addition, environmentally friendly concrete has received increasing attention and applications. This paper summarizes the results of graphene oxide tests in improving the permeability or frost resistance of environmentally friendly concrete (recycled sand concrete and fly ash concrete) in Table 2. It can be found that when the mass proportion of GO mixed exceeds a certain threshold, the agglomeration effect of GO appears, leading to the weakening of the enhancement effect of chloride ion penetration resistance of concrete [74].

When fly ash concrete is mixed with GO of 0.25% mass and 24.3% oxygen, its chloride ion penetration resistance is the strongest [75], and when the recycled sand ultra-high performance concrete is blended with 0.05–0.06% by mass, 50–60% by oxygen, and 0.2–10 μm particle diameter graphene oxide, it has the best chloride ion penetration resistance and frost resistance, and the lowest mass loss rate after 300 cycles of freezing and thawing.

### 4.4. Carbonization Resistance

Generally, the factors affecting concrete carbonization can be divided into three aspects: material factors, environmental factors (carbon dioxide concentration, relative humidity, ambient temperature), and construction factors. Regarding material factors, graphene materials inhibit the carbonation rate of concrete by enhancing its compactness (microscopic pore structure) and alkalinity. Devi [79] exposed concrete samples containing graphene oxide to a sodium sulfate spray in an accelerated carbonation chamber and found that adding graphene oxide can improve the resistance of concrete to sulfate attack, with carbonation depth decreasing as the concentration of graphene oxide percentage increases. He [80] explored the influence of the doped content of GO on the carbonization properties and proposed that when the doped content of GO was 0.05%, the carbonization depth would reach the lowest value.

### 4.5. Fatigue Performance

In terms of bending fatigue, Cho [81] proposed the failure probability of concrete beams under bending fatigue for different mixtures. It has been suggested that GO acts as a buffer during microscopic propagation, reducing creep deformation and crack initiation and propagation. Regarding compression fatigue, Li [82] found that flake graphene slowed down the generation of microcracks in the coupled creep-fatigue phase, delayed the onset of the fatigue phase, and increased the fatigue damage strain. Based on the strain increment in the creep-fatigue coupling stage, a fatigue lifetime prediction equation is derived, which provides a way to estimate the fatigue reliability of graphene concrete structures.

### 4.6. Other Aspects

**Figure 6:**
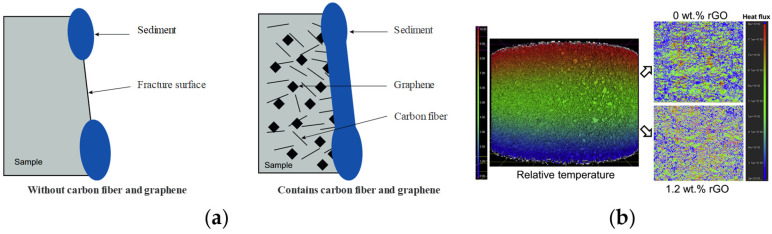
GO assists crack repair and heat dissipation in concrete [86,87]. (**a**) Principle of GO crack repair; (**b**) comparison of temperature nephogram of concrete mixed with RGO.

Researchers have also explored the application of graphene materials in concrete abrasion resistance, antibacterial properties, and crack reinforcement. Liu [83] found that incorporating GO effectively improves fly ash concrete pavement’s crack and abrasion resistance. Li [84] experimented with the biological contamination of graphene concrete and believed that the GO coating significantly reduced the surface energy and roughness of the concrete surface and inhibited the pollution of the concrete surface and growth of marine microorganisms. In addition, graphene materials’ excellent electrical and thermal conductivity provides new possibilities for crack control and self-healing of concrete, as shown in Figure 6. Yang [85] took the internal structure of the reinforced concrete in seawater as the cathode and the platinum electrode as the anode. By using hybrid graphene materials, the electrochemical deposition efficiency is enhanced. The resulting zinc oxide adheres attached to the cracks in the concrete, making the fracture sections more continuous and the structure tighter. Nelyubova [86] was incorporated into GO with good thermal conductivity to effectively disperse heat and control or slow the occurrence of temperature cracks.

## 5. Applications in Other Aspects

### 5.1. Improving the Bonding Force between Material Interfaces

Researchers have used protective coatings or carbon fiber-reinforced composites (CFRP) to enhance the durability and strength of concrete. The introduction of graphene can enhance the bonding between concrete and coating materials, steel bars, and CFRP, thus enhancing the protection and strengthening effect. The following section reviews the application of graphene materials in improving the interfacial bonding between concrete and coating materials, steel reinforcement, and CFRP.

To improve the bonding effect between concrete and CFRP, Al-Saadi [88] used oxidized graphene to synthesize a new type of high-strength self-compacting gel adhesive, which was used to enhance the bond between concrete and CFRP. Through fatigue loading tests, detailed analyses were conducted on failure models [89], microstructure, and local bond-slip relationships [90]. It was found that oxidized graphene improves the elasticity of the gel, resulting in a more homogeneous distribution of the adhesives, CFRP strips, and concrete-bonded surfaces, leading to a significant increase in bond efficiency. Mohammed [91] investigated the impact of high temperatures on the bond strength of GO-reinforced materials. He found that even at high temperatures (800 °C), the gel material still exhibited significant residual bond strength, further expanding the application range of graphene oxide-modified gel adhesives.

**Figure 7:**
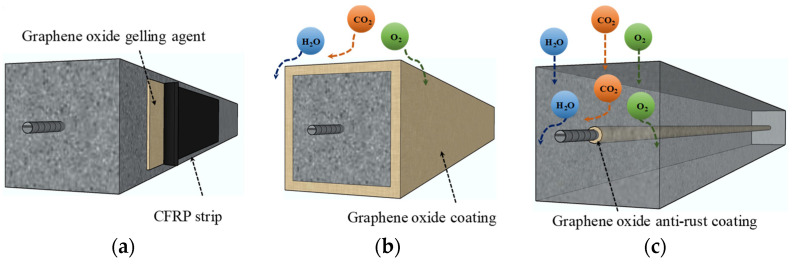
GO improves interfacial adhesion and inhibits corrosion factors from entering the concrete. (**a**) GO gelling agent improves the adhesion between CFRP strip and concrete; (**b**) GO coating inhibits corrosion factors from entering concrete; (**c**) GO anticorrosive coating inhibits corrosion factors from eroding steel bars.

As shown in Figure 7, there are generally two methods for the anti-rust treatment of steel bars: one is to coat the concrete surface with organic coatings to prevent rusting factors (such as oxygen, chloride ions, and acidic gases) from entering the interior of the concrete; another method is to directly coat the surface of the steel bars with an organic anti-corrosion coating to prevent corrosion factors from penetrating the steel bars from the interior of the concrete. Both methods are affected by the bond between the concrete and the protective coating.

In order to improve the adhesion between the concrete and the protective coating, the scholars found that the use of GO to enhance the adhesion is a reliable new approach. Bahraq [92] believed that incorporating graphene materials gave the epoxy resin matrix a suitable configuration (the geometric arrangement of atoms or groups in space caused by the rotation of chemical bonds). At the same time, there is a strong connection between the epoxy molecule and the oxygen-containing functional group on the GO layer, which gives the modified coating excellent adhesion. It is also effective in slowing down the diffusion of water and chloride ions. Sharma et al. [93] modified epoxy-coated steel bars with GO to improve epoxy-coated steel bars’ brittleness and bonding strength. Drawing strength test results proved that the material has stronger muscular bonding to the surrounding concrete, improving the integrity of traditional coated steel bars and concrete. Sun et al. [94] explored the rust prevention principle of graphene-modified steel bar coating materials. They found that graphene materials conduct electricity through the conduction of electrons, extending the permeability channel of the medium and improving the coating’s corrosion resistance. Although the bonding strength between a coated steel bar and concrete is about 15% lower than that of an uncoated steel bar, the overall strength still meets the requirements of the Chinese national standard.

### 5.2. Enhancing the Electrical and Thermal Conductivity of Concrete

Graphene can enhance the electrical and thermal conductivity of cement-based materials, and the modified materials play an essential role in areas such as road snow and ice melting, structural health monitoring, and building energy harvesting, bringing benefits such as reducing energy consumption, improving structural safety, and protecting the environment.

In response to road snow and ice melting, researchers have designed the optimal mixing quantities of graphene materials considering concrete’s mechanical strength and resistivity. Wang et al. [95,96] believe that 10 cm is the best electrode spacing for concrete, and 156 V is the best input voltage. In the case of severe snow disasters in cold regions, multilayer graphene concrete mixed with 0.4% has a resistivity of 12.66 Ω m, making the compressive strength of concrete reach 45.0 MPa, but also can melt 21 cm thick snow in 2 h. In addition to blending single materials to enhance electrical conductivity, Fulham-Lebrasseur [97] considered a composite design of conductive aggregates using copper powder, graphite powder, copper-coated steel fibers, steel fibers, carbon fibers, and graphene. The proposed an optimal blending scheme consisting of 6.0% graphite powder, 0.4% carbon, 1.2% steel, and 0.25% graphene by mass. The resulting improved concrete has a resistivity of 8.9 Ω·m and a compressive strength of 56.8 MPa, with a special de-icing effect and mechanical properties. In addition, Goracci et al. [98] suggested that an ordered C–S–H gel structure around the graphene material also promotes the diffusion of water, another important factor leading to the increase of concrete conductivity.

**Figure 8:**
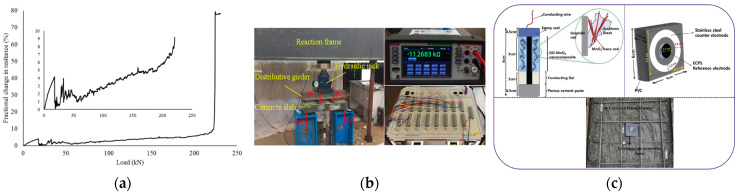
(**a**) Variation of resistance fraction of graphene concrete under different pressures [1]: (**b**) concrete surface strain and crack monitoring based on graphene flexible sensor [103]; (**c**) embedded corrosion rate detection sensor of GO-manganese oxide nanomaterials [104].

Combined with the improved electrical conductivity of the concrete, the researchers also studied the health monitoring of concrete structures. As shown in Figure 8, Song et al. [99] investigated the influence of a few layers of graphene on the piezoresistive properties of concrete and proposed the conductive mechanism of incorporating few-layer graphene into the composite material. Liu et al. [100] established a formula for the response between resistivity and pressure of graphene fiber-reinforced cement mortar. The above studies have laid the theoretical and experimental foundation of graphene sensors for monitoring concrete structures. Later, Rehman et al. [101] monitored the change in the resistance fraction of reinforced concrete beams doped with graphene material under different loads and achieved stress monitoring on reinforced concrete beams. In addition, Jin [102] realized real-time monitoring of the chloride ion penetration degree in mortar paste by measuring the conductivity of graphene cement composite. However, the conductivity of graphene-cement composites is also affected by crack development [103] and pressure changes [100]. Therefore, the accuracy and stability of structural health monitoring using graphene-doped materials needs to be further considered. Wu et al. [103] changed the thinking of previous researchers who used the overall conductive properties of concrete to monitor the structure and innovatively developed a flexible sensor based on the piezoresistive effect of graphene to achieve strain and crack monitoring by attaching it to the concrete surface, with good repeatability of the test results. Karthick et al. [104] made electrochemical corrosion potential and corrosion rate detection sensors using graphene oxide nanomaterials. The sensors were embedded in a reinforced concrete structure, and the measurements showed good stability during the 24 month monitoring period.

### 5.3. Heavy Metal Ion Adsorption and Building Energy Collection

In recent years, the problems of cities’ ecological environment have become worse. With the continuous implementation of the goal of “double carbon” and the continuous promotion of “sponge city” construction, recycled concrete and thermoelectric composite concrete have become a global research hotspot. It is found that graphene-permeable concrete can meet the requirements of light-load road pavement in sponge cities and has good adsorption function for common heavy metal ions (such as lead ions) in water. Zhang Yubin et al. [105] prepared and cured graphene ultra-high performance concrete and found that the material could guarantee excellent mechanical properties and water permeability and had a removal rate of more than 95% of lead ions in the solution. However, the adsorption effect is affected by the pH value, with the highest adsorption rate at pH 4. Muthu et al. [106] prepared porously permeable concrete of various thicknesses using RGO. Despite the exposure of the material to a corrosive acid medium, the removal rate of Cd, Zn, Cu, and Pb from the electroplated wastewater was still as high as 31%. Wijeyawardana et al. [107] concluded that adding RGO effectively improved heavy metal ions’ adsorption efficiency while adding volcanic ash material could reduce the pH value of the effluent from porous concrete.

**Figure 9:**
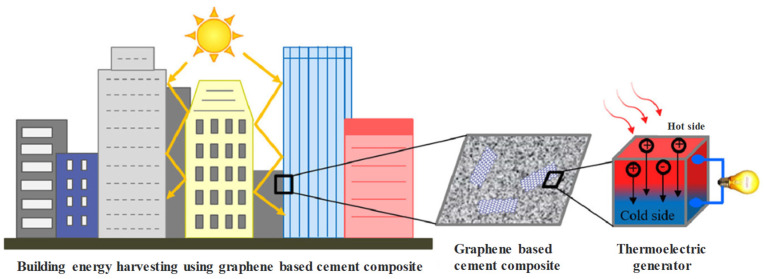
Building energy harvesting based on the thermoelectric effect of graphene-cement composites [100].

In addition, graphene cement-based composite materials can convert the abundant solar energy in concrete structures into electrical energy for energy harvesting in urban buildings. Its principle is shown in Figure 9. Graphene and concrete, two different materials, form a closed loop. When the temperatures of the two nodes are different, a thermoelectric force is created in the loop and an electric current is formed, which enables energy harvesting. Some researchers have mixed other metal materials [108] to improve the thermoelectric effect of graphene concrete [109] and improve energy collection efficiency. Currently, progress is being made in this field [110].

## 6. Discussion

Although graphene and its derivatives have broad research prospects in improving the properties of cement-based materials, some issues and challenges still need to be overcome before they can be generalized to practical engineering applications.

(1) The production cost of graphene materials is high, and it is still challenging to accurately control the size, thickness, number of layers, and oxygen content during the preparation process.

(2) The dispersion of graphene materials is terrible, and the chemical reactivity between cement-based materials and graphene is limited, resulting in a small amount of graphene and its derivatives (the amount of addition in the research paper is no more than 0.3%), which makes it difficult to give full play to its strengthening effect. In addition, the high cost and low efficiency of the commonly used ultrasonic dispersion methods limit their wide application in practical engineering.

(3) The strengthening mechanism of graphene’s different sizes, thicknesses, layers, and oxygen content on cement-based composites is not precise. Researchers have different opinions on the optimal dosage of graphene materials, and no consensus has been reached.

(4) Under the influence of temperature, humidity, alkaline environment, stress, and other factors, the long-term performance stability of graphene and its derivatives in cement has not been fully verified.

## 7. Summary

In this paper, we have summarized and systematically analyzed the research status of graphene and its derivatives in improving the cement base’s operational performance, mechanical properties, durability, and other applications of cement substrates. The conclusions are as follows:

(1) The graphene material adsorbs the free water in the mix, reducing the cement-based material’s fluidity and slowing the concrete’s shrinkage and creep. At the same time, the good thermal conductivity of the graphene materials also promotes hygroscopic rate and heat diffusion in cement-based materials.

(2) There are three main reasons for using graphene to improve the overall mechanical properties of cementitious materials: the structural characteristics of graphene materials give high strength, the ability to promote the hydration reaction of cement, and the ability to fill the cavity gaps. In addition, the oxygen content, diameter, thickness, specific surface area of graphene materials, and the curing time of concrete will indirectly affect the intrinsic mechanical properties and reasonable material dispersion is a prerequisite for improving the overall mechanical properties.

(3) Introducing graphene improves concrete’s impermeability, chloride ion penetration resistance, freeze–thaw resistance, and inhibits fatigue cracks’ expansion. In addition, the antimicrobial, hydrophobic, electrically and thermally conductive properties of graphene materials provide new ideas for the durable protection of marine building structures.

(4) Graphene materials have great potential in improving the bond between cement-based materials, melting snow on a concrete surface, monitoring concrete stress state, absorbing heavy metal ions, and collecting building energy, etc. Continued exploration in these areas will promote the development of concrete structures in the direction of high durability, functionality, intelligence, environmental protection, and energy conservation.

## 8. Future Perspectives

To further advance the engineering application of graphene materials for improving concrete properties, this paper provides the following outlook on the development direction of this field.

(1) Future research should further explore the interaction mechanism between graphene and cement-based materials and develop more diverse graphene derivative materials. Meanwhile, it is necessary to find low-cost and efficient graphene dispersion methods to realize the engineering applications of graphene materials.

(2) To determine the optimal mix range of graphene materials under multiple factors, it is also necessary to conduct systematic tests and simulations to explore the strengthening mechanism of graphene size, thickness, layer number, and oxygen content on cement-based composites.

(3) Further research on the toxicity, environmental impact, and long-term stability of graphene concrete is a vital prerequisite for promoting the widespread use of graphene materials.

(4) Relevant research institutions or departments can also standardize the preparation technology of graphene materials and formulate classification standards for the properties of different graphene materials, which can improve its application effect in cement-based materials and help promote the comparative analysis of different subsequent research results.

## Figures and Tables

**Table 2:** Summary of the effects of different properties and mass ratios of GO on concrete durability.

Durability Enhancement Value		Properties and Mass Ratio of Graphene Oxide		Remarks	Reference
		Material Properties	Mass Content		
Percentage reduction in chloride ion permeability	35.3%	Oxygen accounted for 24.3%	0.25%	When the mass ratio of GO is more significant than 0.25%, the enhancement effect of chloride penetration resistance of fly ash concrete will be weakened.	[75]
	12%	The proportion of oxygen element is less than 53%, the particle diameter is 0.2~10 μm	0.05%	The study object is recycled sand ultra-high performance concrete. When the mass ratio of GO is more significant than 0.05%, the enhancement effect of chloride penetration resistance will be weakened.	[76]
	10.70%	The proportion of oxygen element is more significant than 50%; the particle diameter is 0.2~10 μm	0.06%	The research object is recycled sand ultra-high performance concrete.	[77]
	8.4%	The oxygen component is 56%, the particle diameter is 0.2~10 μm	0.06%	When the dosage is 0.06% and 0.09%, the permeability coefficient of reclaimed concrete decreases by 8.40% and 7.19%, respectively.	[78]
Mass loss rate after 300 cycles of freeze–thaw	0.44%	Oxygen is less than 53%	0.05%	The research object is recycled sand ultra-high performance concrete.	[76]
	0.13%	The proportion of oxygen element is more significant than 50%. The particle diameter is 0.2~10 μm	0.06%	The research object is recycled sand ultra-high performance concrete.	[77]
Relative dynamic modulus of elasticity after 300 cycles of freeze–thaw	98.51%	The proportion of oxygen element is more significant than 50%. The particle diameter is 0.2~10 μm	0.06%	The research object is recycled sand ultra-high performance concrete.	[77]

## Data Availability

Not applicable.
